# Endogenous Glutamate Excites Myenteric Calbindin Neurons by Activating Group I Metabotropic Glutamate Receptors in the Mouse Colon

**DOI:** 10.3389/fnins.2019.00426

**Published:** 2019-05-01

**Authors:** Mathusi Swaminathan, Elisa L. Hill-Yardin, Joel C. Bornstein, Jaime P. P. Foong

**Affiliations:** ^1^Department of Physiology, The University of Melbourne, Parkville, VIC, Australia; ^2^School of Health and Biomedical Sciences, RMIT University, Bundoora, VIC, Australia

**Keywords:** glutamate, synaptic transmission, metabotropic group I glutamate receptors, vGluT2, enteric nervous system, myenteric plexus

## Abstract

Glutamate is a classic excitatory neurotransmitter in the central nervous system (CNS), but despite several studies reporting the expression of glutamate together with its various receptors and transporters within the enteric nervous system (ENS), its role in the gut remains elusive. In this study, we characterized the expression of the vesicular glutamate transporter, vGluT2, and examined the function of glutamate in the myenteric plexus of the distal colon by employing calcium (Ca^2+^)-imaging on Wnt1-Cre; R26R-GCaMP3 mice which express a genetically encoded fluorescent Ca^2+^ indicator in all enteric neurons and glia. Most vGluT2 labeled varicosities contained the synaptic vesicle release protein, synaptophysin, but not vesicular acetylcholine transporter, vAChT, which labels vesicles containing acetylcholine, the primary excitatory neurotransmitter in the ENS. The somata of all calbindin (calb) immunoreactive neurons examined received close contacts from vGluT2 varicosities, which were more numerous than those contacting nitrergic neurons. Exogenous application of L-glutamic acid (L-Glu) and *N*-methyl-D-aspartate (NMDA) transiently increased the intracellular Ca^2+^ concentration [Ca^2+^]_i_ in about 25% of myenteric neurons. Most L-Glu responsive neurons were calb immunoreactive. Blockade of NMDA receptors with APV significantly reduced the number of neurons responsive to L-Glu and NMDA, thus showing functional expression of NMDA receptors on enteric neurons. However, APV resistant responses to L-Glu and NMDA suggest that other glutamate receptors were present. APV did not affect [Ca^2+^]_i_ transients evoked by electrical stimulation of interganglionic nerve fiber tracts, which suggests that NMDA receptors are not involved in synaptic transmission. The group I metabotropic glutamate receptor (mGluR) antagonist, PHCCC, significantly reduced the amplitude of [Ca^2+^]_i_ transients evoked by a 20 pulse (20 Hz) train of electrical stimuli in L-Glu responsive neurons. This stimulus is known to induce slow synaptic depolarizations. Further, some neurons that had PHCCC sensitive [Ca^2+^]_i_ transients were calb immunoreactive and received vGluT2 varicosities. Overall, we conclude that electrically evoked release of endogenous glutamate mediates slow synaptic transmission via activation of group I mGluRs expressed by myenteric neurons, particularly those immunoreactive for calb.

## Introduction

Glutamate is the primary excitatory transmitter in the central nervous system (CNS). But its role the enteric nervous system (ENS) of the gut has remained elusive despite several reports of possible function (Kirchgessner, 2001; Filpa et al., 2016; Seifi and Swinny, 2016).

Several ultrastructural and immunofluorescence studies in rodent models provide evidence for the expression of glutamate, its release and re-uptake transporters, and its various receptor subtypes within the ENS (Liu et al., 1997; Tong et al., 2001; Giaroni et al., 2003; Tsai, 2005; Brumovsky et al., 2011; Seifi and Swinny, 2016). Enteric neurons immunoreactive for glutamate are found in the guinea-pig ileum and are reported to co-express choline acetyltransferase (ChAT), substance P (SP) and/or calbindin (calb) (Liu et al., 1997). Vesicular glutamate transporters (vGluTs) aid in the release of glutamate at pre-synaptic terminals. Although three distinct isoforms of vGluTs (vGluT1, vGluT2, and vGluT3) have been identified in the CNS, vGluT2 is the predominant isoform in the ENS (Tong et al., 2001; Brumovsky et al., 2011; Seifi and Swinny, 2016). While vGluT2 is expressed in enteric varicosities (Tong et al., 2001; Seifi and Swinny, 2016), the targets of these varicosities within the ENS have not been identified. Excitatory levels of glutamate are tightly regulated via excitatory amino acid transporters (EAATs). EAAT is expressed within the ENS, particularly by enteric glia (Liu et al., 1997; Seifi and Swinny, 2016). This is similar to the CNS where EAATs are expressed by glial cells including astrocytes (Rothstein et al., 1994; Lehre et al., 1995). Furthermore, the ionotropic glutamate receptors, *N*-methyl-D-aspartate (NMDA) and α-amino-3-hydroxy-5-methyl-4-isoxazole propionic acid (AMPA), as well as subtypes of metabotropic glutamate receptors (mGluRs) are found on enteric neurons (Broussard et al., 1994; Burns et al., 1994; Liu et al., 1997; Liu and Kirchgessner, 2000; McRoberts et al., 2001; Chen and Kirchgessner, 2002; Tong and Kirchgessner, 2003; Del Valle-Pinero et al., 2007; Foong and Bornstein, 2009; Seifi and Swinny, 2016).

Despite the consensus in the literature about the expression of glutamate and its associated transporters and receptors in the ENS, its role in mediating synaptic transmission remains contentious (Liu et al., 1997; Liu and Kirchgessner, 2000; Ren et al., 2000; Foong and Bornstein, 2009; Wang et al., 2014). In the guinea-pig ENS, NMDA, and AMPA receptors were reported to mediate excitatory post synaptic potentials (EPSPs) in one study (Liu et al., 1997), while a later study found no functional role for ionotropic receptors, but instead demonstrated involvement of slower Group I mGluRs (Ren et al., 2000). Further, pharmacological studies have focused the role of NMDA and AMPA receptors in mediating gut contractility (Luzzi et al., 1988; Shannon and Sawyer, 1989; Wiley et al., 1991; Seifi and Swinny, 2016).

In this study, we examined potential roles of glutamate and its receptors in synaptic transmission within the myenteric plexus. We used calcium (Ca^2+^)-imaging to examine activity of neurons in the myenteric plexus. Connections between glutamatergic terminals and myenteric neurons were examined using high-resolution microscopy and advanced analysis methods. We found that antisera to vGluT2 stain varicosities that contact myenteric neurons. Exogenous application of glutamate excited myenteric neurons by activating glutamate receptors including NMDA receptors, while electrical stimulation evoked responses apparently mediated by group I mGluRs. Most glutamate responsive neurons were calb immunoreactive. Moreover, all calb immunoreactive neurons receive vGluT2 varicosities, thereby suggesting a role for glutamate modulating the excitation of these neurons.

## Materials and Methods

### Experimental Animals

Adult mice (8–15 weeks old) were used. Wnt1-Cre; R26R-GCaMP3 mice of either sex were used in Ca^2+^ imaging experiments. They were the progeny of Wnt1-Cre mice and R2R-GCaMP3 mice (both C57BL/6 background, The Jackson Laboratory) and express the genetically encoded Ca^2+^ indicator, GCaMP3, in all neural crest derived cells including enteric neurons and glia (Danielian et al., 1998; Zariwala et al., 2012; Boesmans et al., 2013). Male C57BL/6 or female R2R-GCaMP3 mice were used for immunohistochemistry studies. All mice were killed by cervical dislocation, as approved by the University of Melbourne Animal Experimentation Ethics Committee. The colon was removed from each mouse and immediately placed in physiological saline (composition in mM: NaCl 118, NaHCO_3_ 25, D-glucose 11, KCl 4.8, CaCl_2_ 2.5, MgSO_4_ 1.2, NaH_2_PO_4_ 1.0) bubbled with carbogen gas (95% O_2_, 5% CO_2_) or in phosphate-buffered saline (PBS). The colon was cut along the mesenteric border, stretched and pinned flat mucosal side up in a Petri dish lined with silicon elastomer (Sylgard 184; Dow Corning, North Ryde, NSW, Australia). The distal colonic region was defined as the area of the colon (3 cm in length) that is 2 cm proximal to the anus.

### Immunohistochemistry

#### Tissue Preparation

Pinned and stretched colonic segments were fixed overnight in 4% formaldehyde in 0.1 M phosphate buffer, pH 7.2, at 4°C, and then cleared of fixative via three washes with PBS. Longitudinal muscle-myenteric plexus (LMMP) whole mount preparations were obtained from the distal colon by microdissection to remove the overlying mucosa-submucosa-circular muscle layers. LMMP preparations were then treated with 0.1% Triton X-100 + 10% CASBLOCK (Invitrogen, Mount Waverley, VIC, Australia; ProSciTech, Thuringowa, QLD, Australia) prior to incubating with a combination of primary antibodies (Table 1) for 24–48 h at 4°C. Preparations were cleared of excess primary antibodies with 3 × 10 min PBS washes, and then incubated with appropriate secondary antibodies (Table 1) for 2.5 h at room temperature. Following another wash with PBS (3 × 10 min), preparations were mounted on a glass slide using a mounting medium (DAKO, Carpinteria, CA, United States).

**Table 1:** Primary and secondary antisera.

Primary antisera	Raised in	Dilution factor	Source
vGluT2	Guinea pig	1:1000	Synaptic Systems
vAChT	Rabbit	1:1000	Synaptic Systems
Synaptophysin	Rabbit	1:100	Abcam
calb	Rabbit	1:1600	SWANT
calr	Goat	1:1000	SWANT
nNOS	Sheep	1:1000	Gift from P. Emson
Hu	Human	1:5000	Gift from Dr. V. Lennon

**Secondary antisera**	**Raised in**	**Dilution factor**	**Source**

Anti-Guinea Pig 594	Donkey	1:400	Jackson Immuno Research Labs
Anti-Guinea Pig FITC	Donkey	1:100	Millipore
Anti-Guinea Pig 647	Donkey	1:200	Jackson Immuno Research Labs
Anti-Rabbit 488	Donkey	1:400	Molecular Probes
Anti-Rabbit 594	Donkey	1:400	Molecular Probes
Anti-Rabbit 647	Donkey	1:400	Molecular Probes
Anti-Sheep 488	Donkey	1:400	Molecular Probes
Anti-Sheep 647	Donkey	1:400	Molecular Probes
Anti-Hu 594	Human	1:750	Jackson Immuno Research Labs

##### Expression of vGluT2 in the myenteric plexus

Longitudinal muscle-myenteric plexus preparations double-labeled for vGluT2 and either one of two synaptic markers (vAChT or synaptophysin) were used to examine the expression pattern of vGluT2 and to determine the degree of co-expression of vGluT2 with the synaptic markers. Tissue samples were viewed using a Zeiss LSM880 Airyscan microscope (Carl Zeiss Microscopy, North Ryde, NSW, Australia). Preparations were labeled with secondary fluorophores 594 (for vGluT2) and 488 (either synaptophysin or vAChT) and were excited with VIS lasers 594 and 488 nm, respectively. Emission was detected using Airyscan filters (BP 420–480 + LP605 for 594 nm and BP 495–550 + LP 570 for 488 nm). Images (1748 pixels × 1748 pixels) were obtained using a Plan-Apochromat 63×/1.40 Oil DIC M27 objective, with a numerical aperture of 1.4, a 1.8× software zoom, z steps of 0.19 μm, 0.60 μs pixel dwell and averaging of 2 using the Zen Black software (Carl Zeiss Microscopy, North Ryde, NSW, Australia). For each combination (vGluT2 and vAChT; vGluT2 and synaptophysin), a total of 3 preparations, each from different animals were examined. In each preparation, 5 images of myenteric ganglia were chosen to be imaged based on their counterstains (vAChT or synaptophysin). The proportion of co-localization between vGluT2 with vAChT or synaptophysin in enteric varicosities was quantified using the image analysis software Imaris 9.0.0 (Bitplane).

The number of vGluT2 immunoreactive varicosities that made close contacts with the cell bodies and dendrites of neuronal nitric oxide synthase (nNOS)+ and calb+ myenteric neurons was examined. Cells positive for nNOS and calb were chosen as they both display characteristic cytoplasmic staining (Mann et al., 1997; Neal and Bornstein, 2007), which facilitates reliable 3D rendering of neuronal surfaces. Additionally, nNOS and calb neurons comprise 35 and 30% of all myenteric neurons in the mouse colon and represent distinct functional classes (Sang and Young, 1996, 1998). High-resolution confocal *z*-stacks were obtained using the Zeiss LSM880 Airyscan microscope (Carl Zeiss Microscopy, North Ryde, NSW, Australia) using parameters described above. For each combination (vGluT2 and nNOS; vGluT2 and calb) a total of 3 preparations, each from different animals were examined. Nine or ten calb or nNOS immunoreactive neurons were examined from each preparation. Imaris software was used to 3D render neuronal and varicosity labeling. We used the distance transformation extension to identify the number of vGluT2 surfaces that were in close contact with each calb and nNOS neurons. This extension creates a pseudo-colored channel, the intensity of this channel indicates the distance between vGluT2 varicosities and 3D rendered neurons of interest. High channel intensity indicates greater distance away from the 3D rendered neuron, and low channel intensity indicates proximity to the 3D rendered neuron. To filter vGluT2 varicosities that were in close contact with 3D rendered neurons of interest, we selected vGluT2 varicosities that located at sites where the pseudo channel intensity was 0 (indicating proximity to the neuron).

##### Proportion of calb and calr neurons in the myenteric plexus

Longitudinal muscle-myenteric plexus preparations were stained for calb, calretinin (calr) and Hu (pan neuronal marker) (Table 1). Immunofluorescently labeled samples were viewed using a LSM Pascal laser scanning microscope (Carl Zeiss Microscopy, North Ryde, NSW, Australia). *Z* stacks were obtained (1024 pixels × 1024 pixels) using a EC Plan-Neofluar 40×/1.30 Oil DIC M27 objective, with a 0.9× software zoom, z-steps of 0.9 μm, 1.60 μs pixel dwell and averaging of 2 using Zen 2.3 (blue edition) software (Zeiss, Australia). A total of 3 preparations were examined, each from a different animal. In each preparation, images of 10 myenteric ganglia were taken based on positive Hu labeling, and at least 250 Hu+ cell bodies were counted. Cell bodies with indistinct faint labeling, likely resulting from auto fluorescence or cross-labeling, were omitted from analysis. The mean proportions of Hu+ neurons immunoreactive for calb, calr or both were determined by calculating the averages from 3 animals.

##### Statistical analysis

All data are represented as mean ± SEM. Unpaired *t*-test analysis was used to compare the proportion of co-localization between vGluT2 with vAChT or synaptophysin (*n* = number of animals examined) and the number of vGluT2 surfaces that were in contact with myenteric neurons (*n* = number of neurons examined). One-way ANOVA was used to compare the mean proportions of Hu+ neurons that label for calb, calr or both (*n* = number of animals examined). All statistical analysis was performed using GraphPad Prism 5.0 (GraphPad Software, San Diego, CA, United States). *P* < 0.05 was considered to be statistically significant.

### Calcium Imaging

#### Tissue Preparation

Distal colon segments were removed from each Wnt1-Cre; R26R-GCaMP mice and placed in Mg^2+^ free physiological saline (composition in mM: NaCl 134 mM, KCl: 3.4 mM, CaCl_2_ 2.8 mM, NaHCO_3_: 16 mM, D-glucose: 7.7 mM; modified from Shannon and Sawyer, 1989). All Ca^2+^-imaging experiments were performed in Mg^2+^ free physiological saline as it has previously been shown in the guinea pig ileum that extracellular Mg^2+^ blocks enteric NMDA receptors so removing Mg^2+^ reveals increased effects of L-glutamate (Luzzi et al., 1988; Shannon and Sawyer, 1989; McRoberts et al., 2011). Colon segments were cut along the mesenteric borders, stretched, and pinned flat mucosal side up in a Sylgard-lined petri dish. Overlaying mucosal and submucosal layers were removed, then the tissues were flipped, and the longitudinal muscle layer was carefully stripped away using fine microdissection forceps to finally obtain preparations of myenteric plexus attached to circular muscle layer (CMMP). The CMMP preparations were stretched over a small inox ring and stabilized by a matched rubber O-ring (Vanden Berghe et al., 2002). A maximum of 3 rings were prepared from each segment of distal colon. The rings were transferred to an organ bath for imaging. The bath was continuously superfused (1 ml/min) with 95% O_2_, 5% CO_2_ Mg^2+^ free physiological saline at room temperature.

#### Imaging and Experimental Protocols

Myenteric ganglia positive for the Ca^2+^ indicator GCaMP3 were imaged (512 pixels × 512 pixels) using a Plan-Apochromat 20×/1,0 DIC (UV) VIS-IR M27 water dipping objective, with a numerical aperture of 1 and a 1× software zoom on an upright Zeiss (Axio Examiner Z.1) microscope with an Axiocam 702 camera (Carl Zeiss Microscopy, North Ryde, NSW, Australia). Images (16 bit) were acquired at 7 Hz. The responses of myenteric neurons to agonists (glutamate, NMDA, AMPA, and GABA) were examined via pressure ejection (spritz, 2 s duration) of each agonist from a micropipette placed at the edge of the imaged ganglion. Up to 5 myenteric ganglia were examined from each animal. At least 3 animals per agonist were investigated unless otherwise stated.

Effects of NMDA receptor antagonist APV on NMDA (100 mM)-evoked and L-Glu (50 mM) evoked [Ca^2+^]_i_ transients was investigated. In each preparation, time controls were first obtained by examining the [Ca^2+^]_i_ transients of myenteric neurons evoked by two spritzes of either agonists (10 min apart) onto a myenteric ganglion in control saline. After this, a different ganglion was chosen and the agonist-evoked [Ca^2+^]_i_ transients of myenteric neurons were examined firstly in control saline, then in the presence of an antagonist following superfusion of the drug for 10 min. Each ringed preparation was only exposed to an antagonist once and up to 4 CMMPs (each from different animals) were investigated per antagonist.

We examined the effects of glutamate receptor antagonists on neurons that displayed both L-Glu (50 mM) spritz- and electrically evoked [Ca^2+^]_i_ transients. Firstly, myenteric ganglia were chemically stimulated via L-Glu (50 mM) spritz as described above. As reported by others (Liu et al., 1997; Kirchgessner, 2001), prolonged and repetitive exposure to L-Glu desensitized GCaMP3+ cells, accordingly the micropipette containing L-Glu was moved away from the ganglia between applications. An interganglionic fiber tract entering the recorded ganglion was electrically stimulated with a single pulse and then a train of pulses (20 pulses, 20 Hz) using a focal stimulating electrode (tungsten wire; 50 μm). The stimuli were applied 1 min apart. Single pulses and trains of 20 pulses (20 Hz) evoke fast and fast-slow EPSPs, respectively (Nurgali et al., 2004; Gwynne and Bornstein, 2007; Foong et al., 2012; Fung et al., 2018). Time controls and antagonist experiments were performed on each preparation as described by Koussoulas et al. (2018). For time controls, the stimulation protocol was performed twice separated by 10 min on a myenteric ganglion in control saline. To test the effects of the antagonists, another ganglion was chosen from the same preparation, and the stimulation protocol was first performed in control saline, then after superfusion of the antagonist into the organ bath for 10 min. Each ringed preparation was only exposed once to an antagonist. A different stimulation protocol was used to examine the effect of the AMPA receptor antagonist CNQX, as only L-Glu-evoked [Ca^2+^]_i_ transients were investigated and only two preparations, each from a different animal, were examined. For all other antagonists, up to 3 CMMPs (each from different animals) were investigated.

Following live-imaging experiments, tissue preparations were fixed overnight with 4% formaldehyde at 4°C and immunostained using primary antisera to the neuron subtype markers nNOS, calb, and calr, or for calb and vGluT2 (Table 1). Imaged ganglia were relocated using an EC Plan-Neofluar 40×/0.75 M27 objective with a numerical aperture of 0.75 on a Zeiss Axio Imager M2 microscope by matching the micrographs with the Ca^2+^ imaging videos. Images were acquired with an Axiocam 506 mono camera using Zen 2.3 (blue edition) software (all from Zeiss, Australia). The immunoreactivity of responding GCaMP3+ neurons to calb, nNOS, or calr was identified.

#### Data Analysis and Statistical Analysis

Analysis was performed using custom-written directives (Li et al., 2019) in IGOR Pro (Wave Metrics, Lake Oswego, OR, United States). Regions of interest were drawn over a selected area of the cytoplasm for each neuron, excluding the nucleus because GCaMP3 is absent from the nuclei (Tian et al., 2009; Yamada and Mikoshiba, 2012). The amplitudes of [Ca^2+^]_i_ transients evoked chemically or electrically were calculated and expressed as the maximum increase in fluorescence from the baseline (Δ*F*_i_/*F*_0_). [Ca^2+^]_i_ transients were only considered if the signal increased above baseline by at least 5 times the intrinsic noise.

For both time control and antagonist experiments, the Δ*F*_i_/*F*_0_ of the second L-Glu spritz or the second electrical stimulation response was normalized and expressed as a fraction of the first (% Δ*F*_i_/*F*_0_). For all L-Glu time control and antagonist experiments consisting of both chemical and electrical stimulations, [Ca^2+^]_i_ transients evoked by electrical stimulation were only analyzed for neurons that previously responded to L-Glu spritz.

At least 3 animals were examined for each experimental set, unless stated otherwise. Data are presented as the mean % Δ*F*_i_/*F*_0_ of the control ± SEM where n = number of neurons examined. Statistical analyses were performed using unpaired *t*-tests with *P* < 0.05 considered statistically significant. Comparisons were performed using GraphPad Prism 5.0 (GraphPad Software, San Diego, CA, United States).

#### Drugs Used

Agonists used included L-Glutamic acid, GABA, *N*-Methyl-D-aspartic acid (NMDA) and Amino-3-hydroxy-5-methylisoxazole-4-propionic acid (AMPA) (all from Sigma-Aldrich, Castle Hill, NSW, Australia). Antagonists used were DL-2-Amino-5-phosphonopentanoic acid (APV) (Sigma-Aldrich), *N*-Phenyl-7-(hydroxyimino) cyclopropa[b]chromen-1a-carboxamide (PHCCC) and 6-Cyano-7-nitroquinoxaline-2,3-dione (CNQX) (from Tocris Bioscience, Avonmouth, Bristol, United Kingdom). All drugs were diluted in distilled water to make stock solutions and in Mg^2+^ free physiological saline on the day of experimentation.

## Results

### vGluT2 Is Mainly Expressed in Non-cholinergic Terminals in the Myenteric Plexus

**FIGURE 1:**
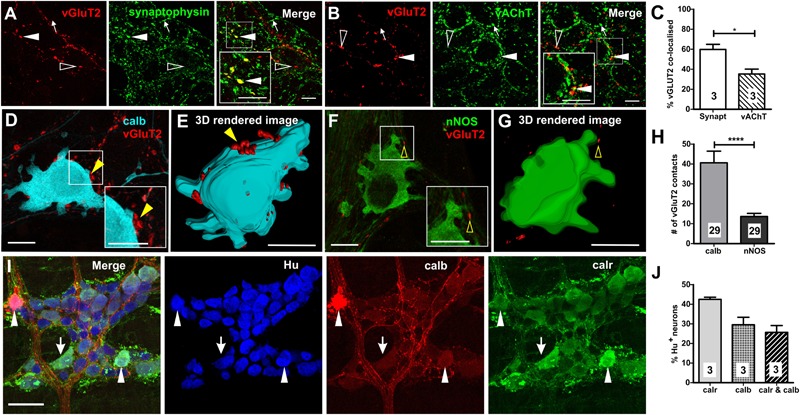
Expression of vGluT2 in the myenteric plexus of mouse distal colon. **(A,B)** High-resolution micrographs illustrating vGluT2 (red), synaptophysin (green), and vAChT (green) immunoreactive varicosities within the myenteric plexus of mouse colon. Scale bars = 10 μm. **(A)** Several vGluT2 immunoreactive varicosities contain synaptophysin (filled arrowhead) but some lack synaptophysin (open arrowhead). Some synaptophysin varicosities lack vGluT2 (arrow). **(B)** Some vGluT2 varicosities contain vAChT (filled arrowhead), but most vGluT2 varicosities lack vAChT (open arrowhead). Likewise, many vAChT varicosities do not express vGluT2 (arrow). **(C)** Histogram showing the percentage of vGluT2 varicosities co-localized with synaptophysin and with vAChT. A significantly higher percentage of vGluT2 varicosities contain synaptophysin compared to those containing vAChT. Numbers on the bar graphs indicate numbers of animals examined. Fluorescence images **(D,F)** and 3D rendered surfaces **(E,G)** of vGluT2 varicosities (red) with a calb+ neuron **(D,E)** (pseudo colored cyan) and a nNOS+ neuron **(F,G)** (green) shows vGluT2 varicosities contacting the calb+ (yellow filled arrowheads) but not the nNOS+ neuron (yellow open arrowheads). All scale bars = 10 μm. **(H)** Histogram illustrating the number of vGluT2 varicosities contacting calb neurons and nNOS neurons. Calb neurons receive significantly more vGluT2 immunoreactive varicosities compared to nNOS neurons. Numbers on the bar graphs indicate numbers of neurons examined. **(I)** Flurorescence micrograph of the myenteric plexus from the distal colon of a C57Bl/6 mice stained for calb (red), calr (green), and pan-neuronal marker Hu (blue). Some calb neurons lack calr (open arrowheads), some calr neurons lack calb (arrows) and some Hu+ neurons contain both calb and calr (filled arrowheads). Scale bar = 50 μm. **(J)** Histogram showing the proportions of calr and/or calb+, Hu+ neurons in the myenteric plexus. Numbers on the bar graphs indicate number of animals examined. ^∗^*p* < 0.05, ^∗∗∗∗^*p*-value < 0.0001; unpaired *t*-test.

We found vGluT2 immunoreactivity in varicosities and terminals, but not in neuronal cell bodies, in the myenteric plexus of the mouse distal colon (Figure 1A,B). This is consistent with previous studies conducted in the rat and mouse oesophageal myenteric plexus (Raab and Neuhuber, 2004, 2005), mouse colorectal (Brumovsky et al., 2011), and colonic myenteric plexuses (Seifi and Swinny, 2016).

To establish the nature of vGluT2 varicosities and terminals in the myenteric plexus, we quantified the co-localization of vGluT2 with two key markers of enteric varicosities (vAChT and synaptophysin). Antisera to synaptophysin (a synaptic vesicle protein) and vAChT (marker of cholinergic varicosities) label many varicosities in the myenteric plexus (Sang and Young, 1998; Sharrad et al., 2013). Most vGluT2 containing varicosities co-expressed synaptophysin (60 ± 5% of vGluT2+ varicosities, Figure 1A,C), but very few synaptophysin+ varicosities contained vGluT2 (5 ± 1% of synaptophysin+ varicosities). Some vGluT2+ varicosities contained vAChT (35 ± 5% of vGluT2+ varicosities, Figure 1B,C), but vAChT varicosities rarely co-expressed vGluT2 (4 ± 1% of vAChT+ varicosities). Thus, only a minority of terminals that release glutamate in the myenteric plexus of the distal colon are likely to be cholinergic.

### vGluT2 Varicosities Innervate Calb Immunoreactive Myenteric Neurons

Examination of close contacts between enteric varicosities and neurons can reveal potential sites of synaptic communication (Mann et al., 1997; Neal and Bornstein, 2007). The number of vGluT2+ close contacts onto two major subtypes of myenteric neurons, calb+ (Figure 1D,E) and nNOS+ (Figure 1F,G) neurons, was quantified to examine whether they receive glutamatergic innervation. Calb+ neurons have either Dogiel type I or II morphology, where type I neurons have elongated cell bodies and lamellar dendrites, while type II neurons have multiple axons and smooth cell bodies (Furness, 2006). Furthermore, Dogiel type I neurons comprise of the interneurons and motor neurons within the enteric circuitry, while type II neurons are typically intrinsic sensory neurons. In this study, most calb+ neurons examined for close contact analysis had Dogiel type I morphology (27/29), the remaining 2/29 had Dogiel type II morphology. The disparity between the two morphological groups was because the outlines of type I neurons were easily distinguishable with high-intensity staining which allowed for 3D rendering of the cell surface, while the staining of the Dogiel type II neurons was weaker and so less suitable for the analysis. All (29/29) calb+ neurons, and 28/29 nNOS+ neurons examined received vGluT2+ innervation. However, calb+ neurons received 3 times as many vGluT2 immunoreactive varicosities compared to nitrergic neurons (calb+ neurons: 41 ± 6, nNOS+ neurons: 14 ± 2 vGluT2+ varicosities; both *n* = 29 neurons, *p* < 0.0001, Figure 1H). Note, the two Dogiel type II calb+ neurons included in the sample had 33 and 43 vGluT2 contacts, well above the mean contacts to nNOS+ neurons. Additionally, the average volume of contacting vGluT2 varicosities was significantly larger for calb+ compared to nNOS+ neurons (calb+: 1.0 ± 0.1 μm^3^, nNOS+: 0.7 ± 0.05 μm^3^; both *n* = 29 neurons, *p* = 0.04, unpaired *t*-test). This suggests that neurally released glutamate plays a greater role in the excitation of calb+ neurons than of nitrergic neurons.

We found both distinct and co-expression of the two Ca^2+^ binding proteins, calb and calr, which is similar to previous studies (Sang et al., 1997; Sang and Young, 1998). Thirty ± 4% (Figure 1I,J) and 43 ± 1% of all Hu immunoreactive myenteric neurons in the distal colon expressed calb and calr, respectively. Of all Hu+ myenteric neurons, 26 ± 4% contain both calb and calr (Figure 1I,J), 17 ± 3% (*n* = 3 animals) only express calr and 4 ± 0.1% only contain calb.

### Many Myenteric Neurons Have Either Glutamate or GABA Receptors or Both

**FIGURE 2:**
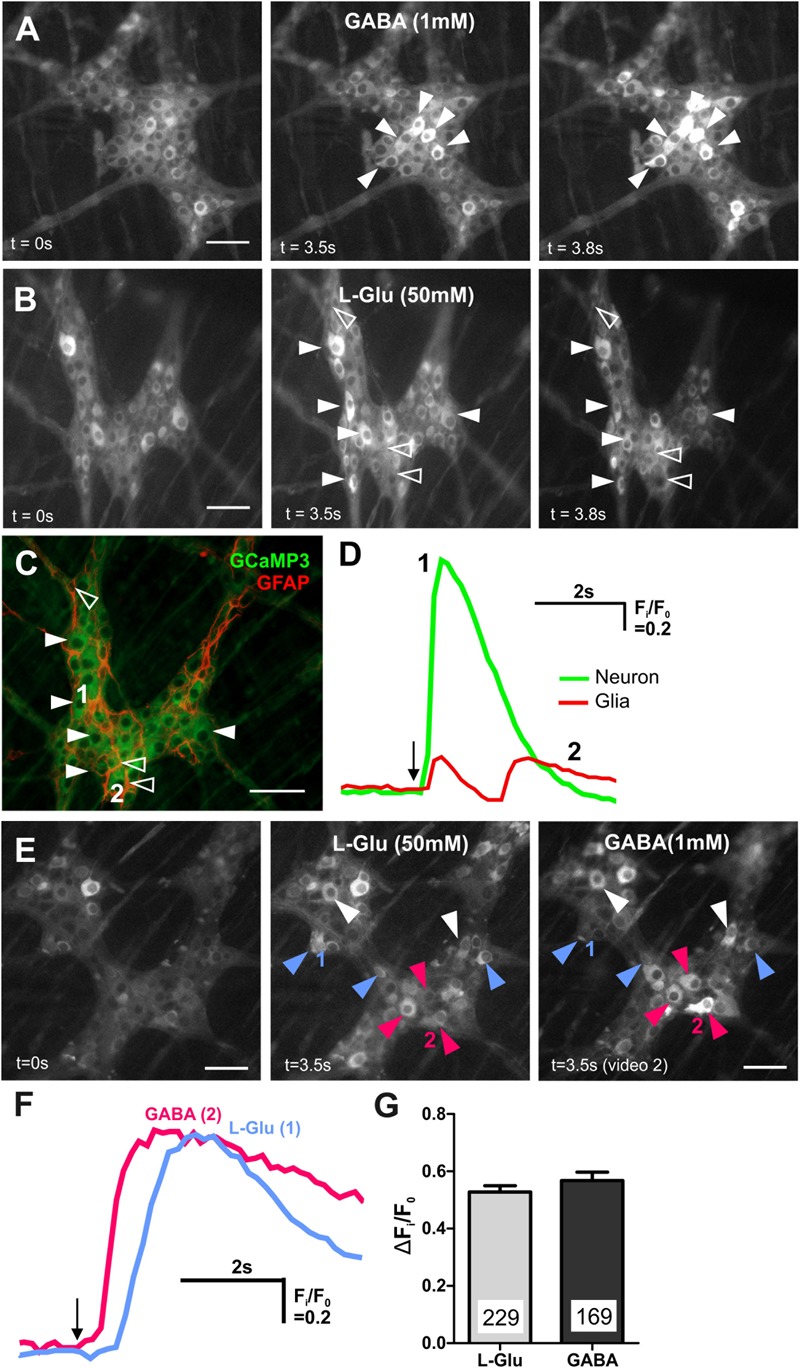
GABA and L-Glu evokes [Ca^2+^]_i_ transients predominantly in different enteric neurons. **(A)** Representative fluorescence micrographs of GABA (1 mM) evoked [Ca^2+^]_i_ transients in myenteric neurons (filled arrowheads) [GCaMP3 signal at rest (*t* = 0 s) and during GABA spritz in neurons (*t* = 3.5 s)]. GABA did not evoke [Ca^2+^]_i_ transients in glia (*t* = 3.8 s) **(B)** fluorescence micrograph of L-Glu evoked [Ca^2+^]_i_ transients in myenteric neurons (filled arrowheads) and glia (open arrowheads) [GCaMP3 signal at rest (*t* = 0s) and during L-Glu stimuation in neurons (*t* = 3.5 s) and in glia (*t* = 3.8 s)]. **(C)** Confocal micrograph of the imaged myenteric ganglion (in **B**) showing GCaMP3+ neurons (filled arrowheads) and GCaMP3+/GFAP+ glia (open arrowheads) that responded to L-Glu spritz. **(D)** Example traces from a neuron (1; green) and a glial cell (2; red) that responded to L-Glu (marked in **C**). The amplitude of [Ca^2+^]_i_ transients were larger in enteric neurons compared to glia. Arrow indicates L-Glu application. **(E)** Representative fluorescence micrographs of L-Glu (50 mM) and GABA (1 mM) evoked [Ca^2+^]_i_ transients in myenteric neurons [GCaMP3 signal at rest (*t* = 0 s) and during L-Glu (*t* = 3.5 s) stimuation and GABA (*t* = 3.5 s) stimulation, respectively]. Most neurons either responded to L-Glu (blue arrowheads) or GABA (pink arrowheads). Few neurons responded to both L-Glu and GABA (white arrowheads). **(F)** [Ca^2+^]_i_ transient traces obtained from neuron 1 and neuron 2 (marked in **E**). Arrow indicates drug application. **(G)** Histogram showing the average amplitude of [Ca^2+^]_i_ transients in response to L-Glu and GABA stimulation. All scale bars = 50 μm. Numbers on the bar graphs indicate numbers of neurons examined.

Central nervous system neurons receive both glutamatergic and GABAergic synaptic inputs. In a previous study, we showed that GABA (1 mM) evokes [Ca^2+^]_i_ transients in over 20% of all GCaMP3+ neurons in the mouse ileum (Koussoulas et al., 2018). For comparison, in the mouse distal colon, we spritzed GABA (1 mM) onto 18 myenteric ganglia and found that this induced robust [Ca^2+^]_i_ transients (Δ*F*_i_/*F*_0_ = 0.60 ± 0.02, *n* = 169 neurons; Figure 2A,F,G) in 23 ± 3% of all GCaMP3+ cells. As in the ileum (Koussoulas et al., 2018), GABA-evoked [Ca^2+^]_i_ transients in the distal colon are most likely to be exhibited exclusively by neurons, as the cell bodies of responding cells were ∼20 μm in diameter consistent with the larger size of enteric neurons compared to glia (Gabella and Trigg, 1984). Further, GABA induced responses were immediate and it is reported that neurons respond instantaneously to stimuli with a sharp increase in [Ca^2+^]_i_, while glia tend to have slower increases in [Ca^2+^]_I_ (Boesmans et al., 2013). L-Glu (50 mM) spritz also evoked robust increases in [Ca^2+^]_i_ in a proportion (26 ± 3%) of all GCaMP3+ cells examined (Δ*F*_i_/*F*_0_ = 0.53 ± 0.02, *n* = 229 neurons, 21 ganglia; Figure 2B–D,F,G). In contrast to GABA, L-Glu spritz also evoked [Ca^2+^]_i_ transients in some GCaMP3+ cells that express the glia marker, glial fibrillary acidic protein (GFAP) (Figure 2B–D) but this was not further investigated in this study. We next recorded the proportion of myenteric neurons that responded to L-Glu (50 mM) and/or GABA (1 mM) (Figure 2E). Seven ganglia (from 2 animals) were spritzed with L-Glu followed by GABA. Of the 392 GCaMP3+ cells examined, 101 (25.8%) responded to L-Glu, 134 (34.2%) to GABA and only 57 (14.5%) had [Ca^2+^]_i_ transients evoked by both L-Glu and GABA. Hence, unlike the CNS, many GCaMP3+ enteric neurons responded to only one of the two amino acids.

As we previously examined GABA-induced responses in mouse enteric preparations, albeit in the distal ileum (Koussoulas et al., 2018), the rest of this study focused on characterizing L-Glu evoked [Ca^2+^]_i_ transients in the mouse distal colon.

### L-Glu Evoked [Ca^2+^]_i_ Transients in Calb+ Myenteric Neurons

**FIGURE 3:**
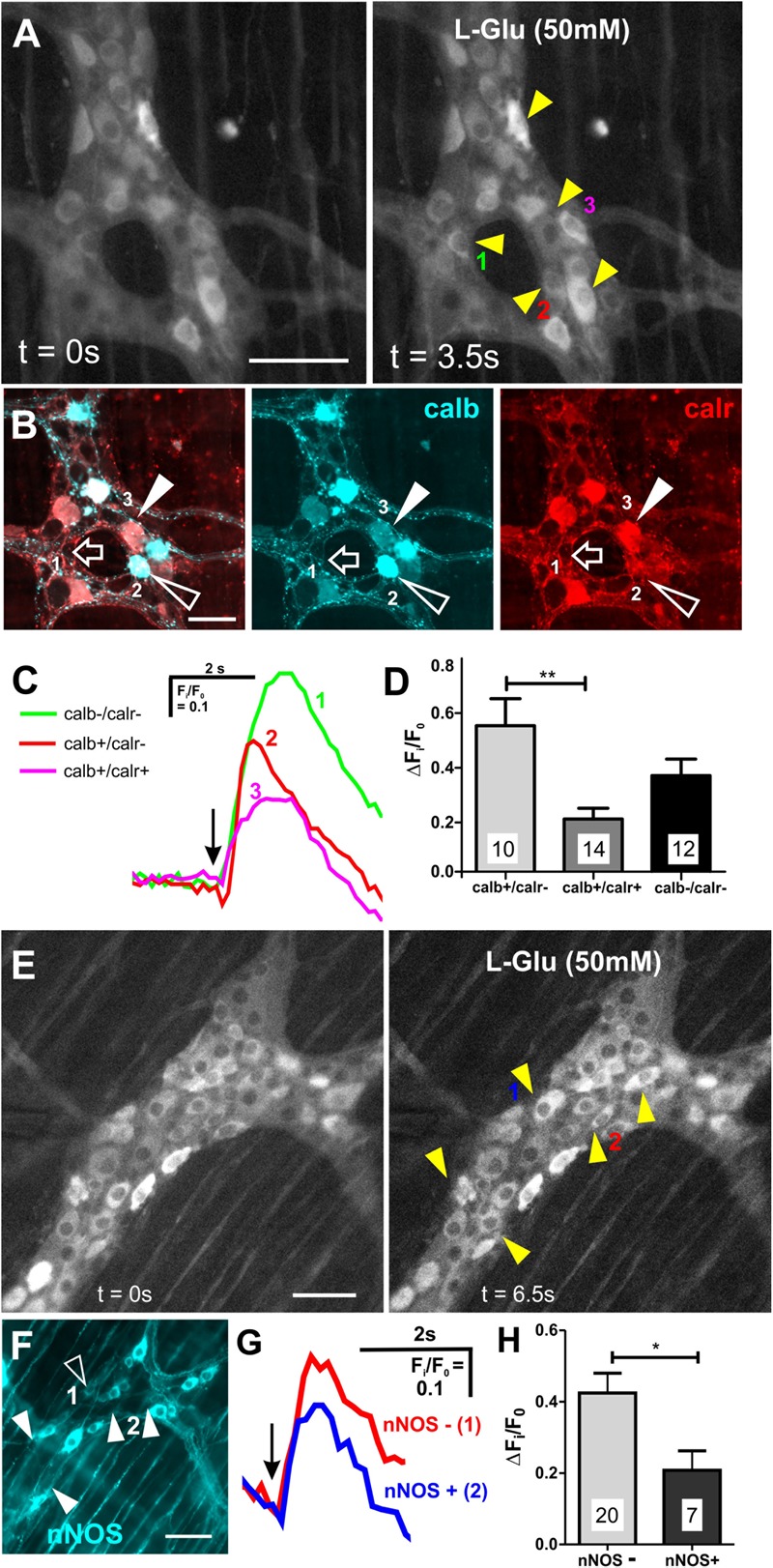
Neurons that expressed calb or lacked nNOS had larger L-Glu-evoked [Ca^2+^]_i_ transients. **(A)** Representative fluroescence micrographs of L-Glu (50 mM) evoked [Ca^2+^]_i_ transients in myenteric neurons [GCaMP3 signal at rest (*t* = 0 s) and during L-Glu stimuation (*t* = 3.5 s)]. **(B)** Confocal micrograph of the imaged myenteric ganglion (in **A**) shows some L-Glu responsive neurons that did not express calb or calr (open arrow, neuron 1), was only immunoreactive for calb (open arrowhead, neuron 2) or was immunoreactive for calb and calr (filled arrowhead, neuron 3). **(C)** Example traces from the 3 neurons (marked in **A,B**) that responded to L-Glu, demonstrating that the neuron that contained calb only exhibited larger L-Glu-evoked [Ca^2+^]_i_ transient. Arrow indicates the when L-Glu was spritzed. **(D)** Histogram showing the average amplitude of L-Glu-evoked [Ca^2+^]_i_ transients. Neurons only positive for calb had larger amplitudes compared to those that contained both calb and calr. **(E)** Representative fluroescence micrographs of L-Glu (50 mM) evoked [Ca^2+^]_i_ transients in myenteric neurons [GCaMP3 signal at rest (*t* = 0 s) and during L-Glu stimuation (*t* = 3.5 s)]. **(F)** Confocal micrograph of the imaged myenteric ganglion (in **A**) shows some L-Glu responsive neurons were nNOS+ (psuedo colored cyan; open arrowheads). Most L-Glu responding neurons lacked nNOS (filled arrowheads). **(G)** Example traces from 2 neurons (marked in **E,F**) that responded to L-Glu, demonstrating that the nNOS – neuron (2) exhibited larger L-Glu-evoked [Ca^2+^]_i_ transient compared to the nNOS+ neuron (1). Arrow indicates when L-Glu was spritzed. **(H)** Histogram showing the average amplitude of L-Glu-evoked [Ca^2+^]_i_ transients. nNOS– neurons had larger amplitudes compared to nNOS+ neurons. All scale bars 50μm. Number of neurons examined are indicated on the bar graphs; ^∗^*p* < 0.01, ^∗∗^*p* = 0.003; unpaired *t*-test.

Most neurons (85/146, 58%) that responded to L-Glu (50 mM) were immunoreactive for calb. The amplitude of the L-Glu response was similar in neurons that either expressed or lacked calb (calb+ Δ*F*_i_/*F*_0_: 0.5 ± 0.03, *n* = 85 neurons; calb- Δ*F*_i_/*F*_0_: 0.5 ± 0.03; *n* = 61 neurons). Calb staining co-localized with calr in two preparations. Of the 36 L-Glu responders in these preparations, 14 were calb+/calr+, 11 were calb-/calr-, 10 were calb+ only, and 1 neuron was calr+ only (Figure 3A,B). Calb+ neurons that responded to L-Glu included both Dogiel types I and II neurons. The majority of calr+ neurons in the ganglia examined did not respond to L-Glu (68%). The amplitudes of responses in neurons that expressed both calb and calr were significantly lower than in neurons that only contained calb (calb+/calr+ Δ*F*_i_/*F*_0_: 0.19 ± 0.03, *n* = 14 neurons; calb+ only Δ*F*_i_/*F*_0_: 0.53 ± 0.10, *n* = 10 neurons, *p* = 0.003, One-way ANOVA, Figure 3C,D). No significant differences in the amplitude of Ca^2+^ responses were observed between the other groups identified. These findings suggest that glutamate has a greater influence on the excitation of neurons that are immunoreactive for calb only and not calr.

A large proportion of L-Glu responsive neurons (61/146) did not express calb, so we examined immunoreactivity of these neurons for nNOS, which marks another major subpopulation of myenteric neurons (Figure 3E,F). Only 7/27 (26%) L-Glu-responding neurons were nNOS+ and the amplitude of this response was significantly lower than in neurons without nNOS (nNOS+ Δ*F*_i_/*F*_0_: 0.2 ± 0.05, *n* = 7 neurons; nNOS- Δ*F*_i_/*F*_0_: 0.4 ± 0.06, *n* = 20 neurons, *p* = 0.04, Figure 3E–H). This suggests that glutamate does not have a major influence in exciting nNOS+ neurons.

### Although NMDA Receptors Are Expressed by L-Glu Responding Neurons, They Are Not Involved in Glutamatergic Synaptic Transmission

**FIGURE 4:**
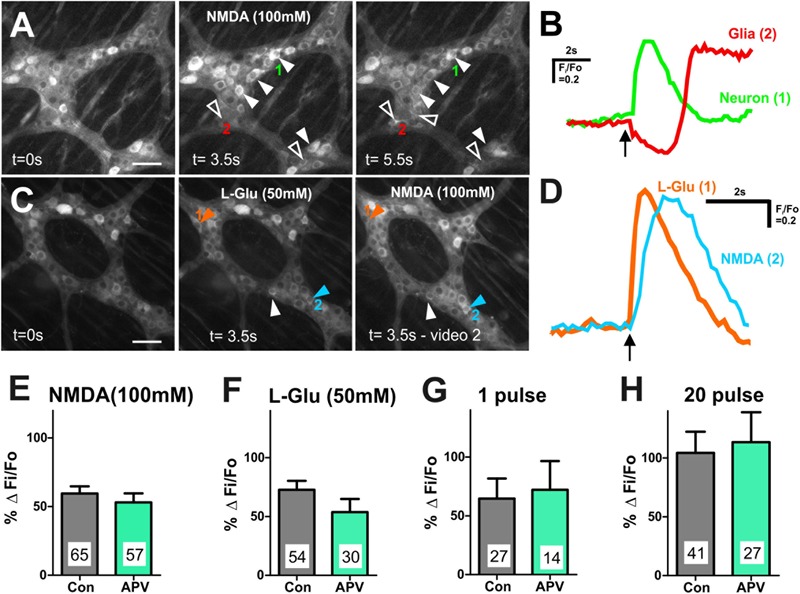
Many myenteric neurons exhibited [Ca^2+^]_i_ transients in response to L-Glu and NMDA. **(A)** Representative fluorescence micrographs of NMDA (100 mM) evoked [Ca^2+^]_i_ transients in myenteric neurons (filled arrowheads) and glia (open arrowheads) [GCaMP3 signal at rest (*t* = 0 s) and during NMDA spritz in neurons (*t* = 3.5 s) and in glia (*t* = 5.5 s)]. **(B)** Example traces from a neuron (1) and a glial cell (2; marked in **A**) that responded to L-Glu. Glial cell shows delayed response to NMDA compared to the GCaMP3+ neuron. Arrow indicates NMDA application. **(C)** Representative fluorescence micrographs of L-Glu (50 mM) and NMDA (100 mM) evoked [Ca^2+^]_i_ transients in myenteric neurons [GCaMP3 signal at rest (*t* = 0 s) and during L-Glu stimulation and NMDA stimulation, respectively]. Most L-Glu responding neurons also respond to NMDA (filled arrowhead). Some neurons either respond to L-Glu (orange arrowhead) or NMDA (blue arrowhead). **(D)** [Ca^2+^]_i_ transient traces obtained from neuron 1 and neuron 2 (marked in **C**). Arrow indicates drug application. Histograms showing pooled [Ca^2+^]_i_ transient amplitudes from all neurons stimulated with NMDA **(E)** and L-Glu **(F)**, and from L-Glu responding neurons stimulated with 1 pulse **(G)** and 20 pulse **(H)** in time control (con) and in the presence of APV. Changes in amplitude after application APV are presented as a percentage of the first response in control saline (% Δ *F*_i_/*F*_0_). APV did not alter the amplitude of agonist or electrically evoked [Ca^2+^]_i_ transients in myenteric neurons. All scale bars 50 μm. Number of neurons examined are indicated on the bar graphs.

Ionotropic glutamate receptors and their subunits are present on guinea-pig enteric neurons, where they have been reported to mediate glutamatergic neurotransmission (Liu et al., 1997; Kirchgessner, 2001). We found that spritzed NMDA (100 mM) induced an increase in [Ca^2+^]_i_ in 22 ± 3% of GCaMP3+ cells examined (Δ*F*_i_/*F*_0_ = 0.5 ± 0.04, *n* = 95 neurons, 10 ganglia; Figure 4A,B). Like L-Glu, NMDA spritz evoked delayed [Ca^2+^]_i_ transients in some GCaMP3+ glia (Figure 4A,B), but the glial responses were not investigated further. L-Glu (50 mM) was applied to 4 myenteric ganglia (from 1 animal) followed by NMDA to examine if neurons respond to both L-Glu and NMDA (Figure 4C,D). Of the 212 GCaMP3+ cells examined, 39 (18%) responded to L-Glu, 29 (14%) responded to NMDA, and 17 (8%) responded to both L-Glu and NMDA. Hence, most (17/29, 59%) NMDA responding GCaMP3+ cells also responded to L-Glu. Many L-Glu responding GCaMP3+ cells (22/39, 56%) did not respond to NMDA, probably because other glutamate receptors were present. Likewise, some (12/29, 41%) NMDA responding GCaMP3+ cells did not respond to L-Glu, indicating that there may be non-specific actions of NMDA on myenteric neurons.

We examined the effects of the NMDA receptor antagonist APV (100–500 μM) on [Ca^2+^]_i_ transients evoked by exogenous NMDA and L-Glu. APV significantly reduced the proportion of GCaMP3+ cells that responded to NMDA (100 mM) (Fisher’s exact test *p* = 0.023, Table 2), but had no effect on the amplitude of NMDA-evoked [Ca^2+^]_i_ transients (% Δ*F*_i_/*F*_0_ control: 59.5 ± 5.2, *n* = 65 neurons; APV: 53.1 ± 6.6, *n* = 57 neurons; Figure 4E). Similarly, APV significantly reduced the number of L-Glu responding neurons (Fisher’s exact test *p* = 0.02, Table 2), but not the amplitude of the L-Glu- evoked [Ca^2+^]_i_ transients (% Δ*F*_i_/*F*_0_ control: 73 ± 8, *n* = 54 neurons; APV: 54 ± 11, *n* = 30 neurons; *p* = 0.2; Figure 4F).

**Table 2:** Number of responding neurons during time controls and in the presence antagonists.

	Time	Time
Stimulation/antagonist	control (1)	control (2)	Control	Antagonist
NMDA (100 mM) spritz/APV (100 μM)	65	54/65	57	37/57**^∗^**

L-Glu (50 mM) spritz/APV (500 μM)	54	47/54	30	19/30**^∗^**
1 pulse/APV (500 μM)	27	14/27	21	14/21
20 pulse/APV (500 μM)	41	29/41	27	17/27

L-Glu (50 mM) spritz/CNQX (20 μM)	25	18/25	38	30/38

L-Glu (50 mM) spritz/PHCCC (30 μM)	75	60/75	61	41/61
1 pulse/PHCCC (30 μM)	35	24/35	37	15/37
20/PHCCC (30μM)	62	56/62	50	37/50**^∗^**

*^∗^P < 0.05 Fisher’s Exact Test*.

Inputs to myenteric ganglia were electrically stimulated to investigate the effects of APV on potential endogenous glutamate neurotransmission. APV (500 μM) did not affect 1 pulse- (% Δ*F*_i_/*F*_0_ control: 65 ± 17%, *n* = 27 neurons; % Δ*F*_i_/*F*_0_ APV: 72 ± 25%, *n* = 14 neurons; *p* = 0.8; Figure 4G) or 20 pulse-evoked (% Δ*F*_i_/*F*_0_ control: 104 ± 18, *n* = 41 neurons; % Δ*F*_i_/*F*_0_ APV: 114 ± 25%, *n* = 27 neurons; *p* = 0.8; Figure 4H) [Ca^2+^]_i_ transients in L-Glu responding neurons. Further, APV did not affect the number of neurons that exhibited electrically evoked [Ca^2+^]_i_ transients relative to time controls (Table 2).

We applied AMPA (50 and 100 μM) to some myenteric ganglia using concentrations effective in other studies (Parsons et al., 1994; Nissen et al., 1995), but it did not evoke reliable responses. Further, the AMPA receptor antagonist CNQX (20 μM) did not alter the number of neurons that exhibited L-Glu evoked [Ca^2+^]_i_ transients relative to time controls (Table 2) or their amplitude (% Δ*F*_i_/*F*_0_ control: 69 ± 12, *n* = 25 neurons; CNQX: 87 ± 17, *n* = 38 neurons; *p* = 0.4). Thus, AMPA receptors were not investigated further.

### Glutamatergic Synaptic Transmission Involves Group I mGlu Receptors in Myenteric Ganglia

Group I metabotropic glutamate receptors (mGluRs) are involved in enteric neurotransmission in guinea pig (Ren et al., 2000; Foong and Bornstein, 2009). We focused on neurons that expressed glutamate receptors by first identifying the neurons that responded to exogenous L-Glu (50 mM). However, it should be noted that L-Glu responsive neurons may include secondary neurons that do not express glutamate receptors themselves but receive synaptic input from other neurons that do. We examined whether the Group I mGluR antagonist PHCCC (30 μM) affects the L-Glu- and electrically evoked [Ca^2+^]_i_ transients of these neurons to determine whether they express Group I mGluRs, and if the probable release of endogenous glutamate in response to electrical stimuli can activate these receptors.

**FIGURE 5:**
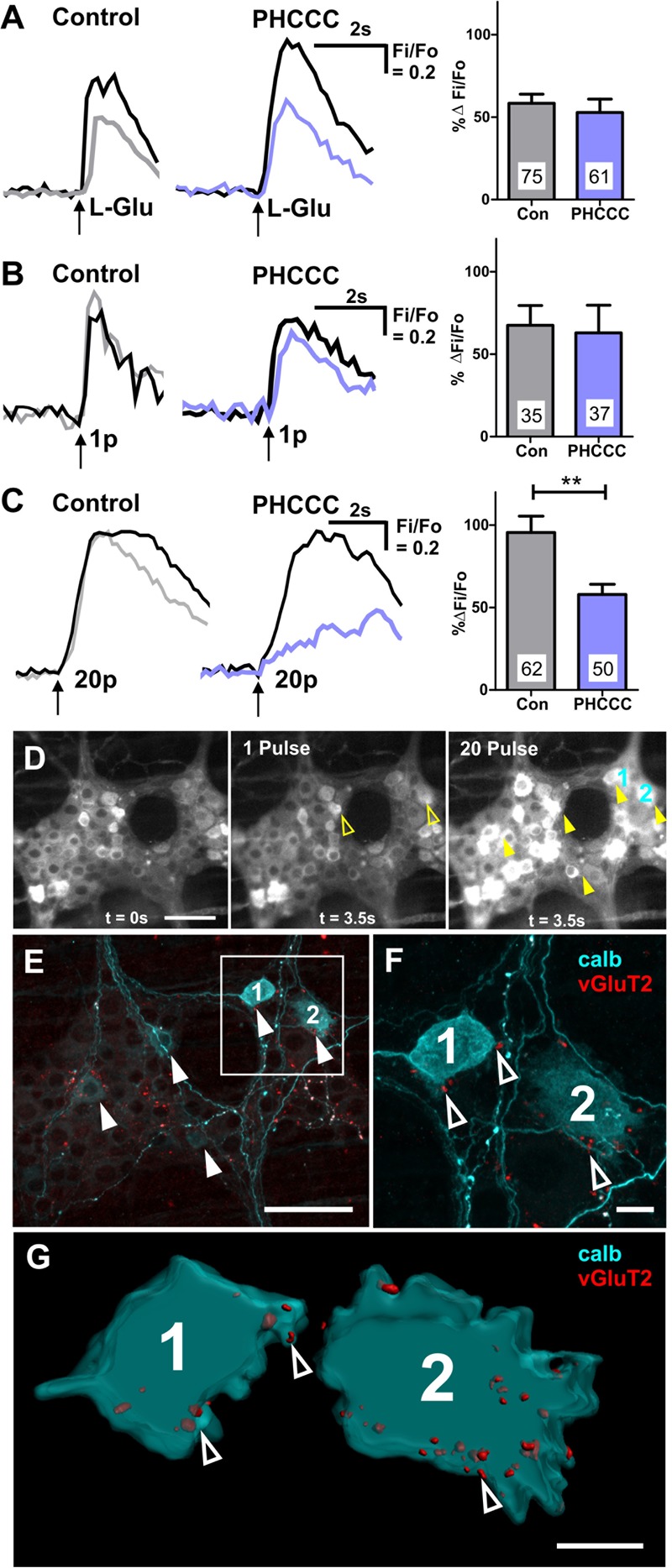
Calbindin+ myenteric neurons receive vGluT2+ varicosities, and exhibit 20 pulse-evoked [Ca^2+^]_i_ transients that are mediated by Group I mGluRs. Representative traces of [Ca^2+^]_i_ transients evoked by L-Glu (50 mM) spritz **(A)**, 1 pulse **(B)**, and 20 pulse **(C)** electrical stimulation during time controls (1st stimulation: black trace; 2nd stimulation: gray trace) and PHCCC treatment (control: black trace; PHCCC: purple). Histograms showing pooled [Ca^2+^]_i_ transient amplitudes from all neurons stimulated with L-Glu **(A)**, 1 pulse **(B)**, and 20 pulse **(C)** in time control (con) and in the presence of PHCCC. Changes in amplitude after application PHCCC are presented as a percentage of the first response in control saline (% Δ*F*_i_/*F*_0_). Numbers on bar graphs indicate the number of neurons examined. PHCCC significantly reduced the amplitude of 20 pulse-evoked [Ca^2+^]_i_ transients in L-Glu responding neurons (^∗∗^*p* < 0.01; unpaired *t*-test). **(D)** Representative fluroescence micrographs of 1 pulse and 20 pulse electrical stimulation-evoked [Ca^2+^]_i_ transients in myenteric neurons [GCaMP3 signal at rest (*t* = 0 s) and during 1 pulse and 20 pulse electrical stimulation stimuation (*t* = 3.5 s), respectively]. **(E)** Confocal micrograph of the imaged myenteric ganglion (in **D**) stained for calb. Some neurons calb+ neurons responded to 1 pulse (yellow open arrows in **D**), most calb+ neurons identified responded to 20 pulse stimulations (yellow filled arrows in **D**). Scale bars 50 μm. Enlarged confocal micrograph **(F)** and 3D rendered surfaces **(G)** of a section in **(E)**, illustrate close contacts of vGluT2 varicosities (open arrowheads) onto two calb+ neurons (marked 1 and 2) that responded to 20 pulse stimulation. Scale bar 10 μm.

PHCCC did not alter the proportion of GCaMP3+ cells that responded to L-Glu (Table 2) or the amplitude of L-Glu evoked [Ca^2+^]_i_ transients (% Δ*F*_i_/*F*_0_ control: 59 ± 6, *n* = 75 neurons; % Δ*F*_i_/*F*_0_ PHCCC: 53 ± 8, *n* = 61 neurons; Figure 5A). PHCCC also did not affect the proportion of responsive GCaMP3+ cells (Table 2) or amplitude of [Ca^2+^]_i_ transients evoked by 1 pulse stimulation (% Δ*F*_i_/*F*_0_ control: 68 ± 12%, *n* = 35 neurons; % Δ*F*_i_/*F*_0_ PHCCC: 63 ± 17%, *n* = 37 neurons; *p* = 0.828, Figure 5B). However, it significantly reduced the proportion of neurons that responded to 20 pulse stimulation (*p* = 0.04, Fisher’s exact test; Table 2) and the amplitude of the 20 pulse-evoked [Ca^2+^]_i_ transients (% Δ*F*_i_/*F*_0_ control: 96 ± 10, *n* = 62 neurons; % Δ*F*_i_/*F*_0_ PHCCC: 58 ± 6, *n* = 50; *p* = 0.003, Figure 5C).

*Post hoc* immunohistochemistry was conducted to identify the neurochemistry of the responding neurons. Of the 49 neurons that exhibited 1 pulse-evoked [Ca^2+^]_i_ transients, 24 (49%) were immunoreactive for calb+, while 39/73 (53%) of the neurons that exhibited 20 pulse-evoked [Ca^2+^]_i_ transients were immunoreactive for calb. In a particular ganglion examined, 6 of the 17 neurons that responded to 20 pulse stimulation were calb+ and of these 5 had vGluT2+ varicosities contacting their somata (Figure 5D–G). Moreover, the 20 pulse responses in the calb+ neurons in this ganglion were depressed by the group I mGluR antagonist. Thus, calb+ myenteric neurons receive glutamatergic innervation and receive slow synaptic inputs mediated by endogenous release of glutamate activating group I mGluRs.

PHCCC was dissolved in DMSO, so vehicle control experiments were conducted to eliminate the possibility of DMSO effects on [Ca^2+^]_i_ transients. DMSO did not affect the proportion of L-Glu responders (Fisher’s exact test *p* = 0.6, Table 2) or the amplitude of L-Glu-evoked [Ca^2+^]_i_ transients (% Δ*F*_i_/*F*_0_ control: 94 ± 12%, *n* = 20 neurons; % Δ*F*_i_/*F*_0_; DMSO: 79 ± 7%, *n* = 35 neurons; *p* = 0.3). DMSO also did not affect the numbers of responsive neurons that responded to 1 pulse or 20 pulse stimulation (both *p* > 0.3 Fisher exact test, Table 2) or the amplitude of electrically evoked [Ca^2+^]_i_ transients (% Δ*F*_i_/*F*_0_ 1 pulse control: 158 ± 36%, *n* = 15 neurons; % Δ*F*_i_/*F*_0_ DMSO: 124 ± 35%, *n* = 20 neurons; *p* = 0.5; 20 pulse % Δ*F*_i_/*F*_0_ control: 104 ± 14%, *n* = 17 neurons; % Δ*F*_i_/*F*_0_ DMSO: 114 ± 17%, *n* = 30 neurons; *p* = 0.7).

## Discussion

How glutamate mediates synaptic transmission in the ENS has been difficult to decipher even though several studies implicate glutamate in the regulation of gastrointestinal function (Kirchgessner, 2001; Filpa et al., 2016). This may be due to its role as an auxiliary neurotransmitter compared to the dominant neurotransmitter acetylcholine, and to the vast diversity of glutamatergic receptors expressed in this system (Liu et al., 1997; Ren et al., 2000; Kirchgessner, 2001; Seifi and Swinny, 2016). Here, we provide several lines of evidence for glutamatergic excitation of a proportion of calb immunoreactive neurons via activation of Group I mGluRs. Firstly, we show that most vGluT2+ varicosities are non-cholinergic and many express synaptophysin. We also show that vGluT2+ varicosities preferentially surround calb+ neurons rather than nitrergic neurons. Likewise, we found that calb+ neurons are excited by glutamate receptors. Further, we showed that electrically evoked release of putative endogenous glutamate contributes to slow synaptic transmission in myenteric neurons, especially those immunoreactive for calb, via the activation of Group I mGluRs.

### vGluT2+ Varicosities Are Often Non-cholinergic and Contain the Synaptic Vesicle Protein Synaptophysin

Although vGluT2 expression is observed in varicosities within the myenteric plexus of several rodent models, the neurochemistry of glutamate containing varicosities and their innervation patterns have not been examined in detail (Liu et al., 1997; Tong et al., 2001; Brumovsky et al., 2011; Seifi and Swinny, 2016). Our observation of vGluT2 expression in myenteric varicosities of mouse is consistent with previous studies (Tong et al., 2001; Brumovsky et al., 2011; Seifi and Swinny, 2016). Varicosities are putative neurotransmitter release sites in the enteric neural circuitry. Using advanced imaging and quantification methods adapted for enteric preparations, we found that most vGluT2+ varicosities (∼65%) are non-cholinergic as they lacked vAChT, confirming previous qualitative descriptions (Tong et al., 2001; Seifi and Swinny, 2016). We found that only a small proportion of few vGluT2+ varicosities contain vAChT. These varicosities are likely to originate from intrinsic glutamatergic enteric neurons or from dorsal root ganglion neurons (Liu et al., 1997; Tong et al., 2001). The non-cholinergic vGluT2+ varicosities may also originate from extrinsic neurons, as previous studies have reported the expression of vGluT2+ neurons in the nodose and dorsal root ganglia, which innervate the mouse stomach (Tong et al., 2001). In addition, we found that most vGluT2+ varicosities (60%) co-express the synaptic vesicle protein synaptophysin, a protein involved in exo- and endocytosis of synaptic vesicles in the CNS (Edelmann et al., 1995;Takamori et al., 2006; Kwon and Chapman, 2011). Thus, potential glutamate release sites are present within the myenteric plexus, and synaptophysin, a major constituent of glutamatergic synaptic vesicles in the CNS (Takamori et al., 2006), is most likely involved in regulating glutamate release in the ENS. However, given that some vGluT2+ varicosities lack synaptophysin, other families of synaptic vesicles are probably involved in endo- and exocytosis of glutamate within the ENS.

### Glutamate Excites Calb+ Myenteric Neurons by Activating Glutamate Receptors Including NMDA and Group I mGluRs

Exogenous application of L-Glu evoked [Ca^2+^]_i_ transients in some myenteric neurons, these neurons were largely distinct from those that responded to the other prominent CNS neurotransmitter, GABA. Most L-Glu responders were calb+, some of which co-express calr, but fewer L-Glu responders were nNOS+, indicating that functional glutamatergic receptors are predominantly localized to calb+ myenteric neurons. These findings align with our anatomical observations that all calb+ neurons examined received vGlu2+ varicosities and are contacted by substantially more vGluT2+ varicosities than nNOS+ neurons. L-Glu responding calb+ neurons exhibit either Dogiel types I and II morphology. In combination with our finding that all Dogiel type I calb+ neurons examined receive vGluT2 innervation, we provide strong evidence that glutamate excites calb+ interneurons. Though under-represented, our data also suggests that Dogiel type II, calb+ neurons which are typical intrinsic sensory neurons also receive functional glutamatergic inputs. Moreover, the L-Glu responsive neurons that co-express calb and calr are probably either intrinsic sensory and interneurons, as calb+ varicosities do not innervate the smooth muscle layers (Sang and Young, 1998). Most calr+ only and nNOS+ neurons were unresponsive to L-Glu, suggesting that glutamate does not influence the excitation of motor neurons, interneurons and some intrinsic sensory neurons identified by these markers. Therefore, these observations indicate that glutamate is likely to play a significant role in modulating the activity of neural pathways involving calb+ interneurons and intrinsic sensory neurons.

Thus far, electrophysiological studies have resulted in conflicting findings in relation to the involvement of NMDA receptors in mediating glutamate-evoked depolarizations within the ENS (Liu et al., 1997; Ren et al., 2000). Liu et al. (1997) observed that the NMDA receptor antagonist AP5 inhibited glutamate-elicited slow depolarizations in enteric neurons. However, Ren et al. (2000) reported that glutamate induced excitation was unaffected by the NMDA receptor antagonists MK-801 and D-APV. In this study, we found that the amplitudes of NMDA- and L-Glu-evoked [Ca^2+^]_i_ transients were unaffected by APV, but that APV reduced the number of neurons that respond to NMDA and L-Glu.

Although our findings align with previous reports that identify expression of NMDA receptors in enteric neurons (Broussard et al., 1994; Burns et al., 1994; Liu et al., 1997; McRoberts et al., 2001; Del Valle-Pinero et al., 2007), the lack of effect of NMDA receptor blockade could be due to the presence of other glutamate receptors such as Kainate receptors, which have also been shown to be expressed on myenteric neurons (Carpanese et al., 2014). Likewise, [Ca^2+^]_i_ transients are dominated by Ca^2+^ entry due to action potentials (Vanden Berghe et al., 2000), therefore it is likely that the amount of Ca^2+^ into these neurons was insufficient to evoke depolarizations. It is also possible that the lack of response observed in some neurons during NMDA blockade could be due to the high-density of NMDA receptors on those particular neurons. Indicating potential heterogeneity in the expression of NMDA receptors on myenteric neurons. Therefore, further clarification into the expression of NMDA receptor subunits within the myenteric plexus is required. Additionally, it should be noted that the concentration (100 mM) of NMDA in the spritz pipette required to evoke [Ca^2+^]_i_ transients in this study is higher than the concentration (10 mM) used in a previous study which examined membrane potentials of neurons with intracellular recording (Liu et al., 1997), thus, we cannot exclude the possibility of potential non-specific effects of the agonist in this study.

### Calb+ Myenteric Neurons Receive Slow Synaptic Transmission Mediated by Group I mGluRs

Activation of NMDA, AMPA, and Group I mGlu receptors depolarizes enteric neurons in guinea pig tissues (Liu et al., 1997; Liu and Kirchgessner, 2000; Ren et al., 2000; Foong and Bornstein, 2009), but the precise involvement of these receptors in synaptic transmission is unclear. We used a single pulse and a train of electrical pulses as stimuli, which evoke fast, and fast-slow EPSPs in mouse myenteric neurons (Shuttleworth and Smith, 1999; Nurgali et al., 2004; Gwynne and Bornstein, 2007; Foong et al., 2012, 2015; Koussoulas et al., 2018) to ascertain the effects of potential endogenous glutamate release on L-Glu–responsive neurons. The amplitudes of both electrically evoked [Ca^2+^]_i_ transients, and the number of neurons responding to electrical stimuli were unaffected by the competitive NMDA receptor antagonist, APV. Thus, NMDA receptors are unlikely to mediate fast and slow EPSPs in mouse myenteric neurons, as previously reported for guinea pig (Liu et al., 1997; Ren et al., 2000). Nevertheless, Liu et al. (1997) found that APV reduced the amplitude of glutamate-evoked slow depolarizations in guinea pig enteric neurons but suggested that these slow responses are unlikely to represent slow EPSPs and that NMDA receptors play a modulatory role in the enteric neural circuitry. NMDA receptors are non-specific cation channels (Hansen et al., 2018), and increase underlying membrane conductance, but slow EPSPs in the ENS are associated with decreases in membrane conductance (Gwynne and Bornstein, 2007). Indeed activation of NMDA receptors would produce [Ca^2+^]_i_ transients in the absence of action potentials. Thus, even a subthreshold NMDA response might mimic an action potential driven [Ca^2+^]_i_ transient. The antagonist used in this study acts on all four GluN2 subunits of the NMDA receptor, so the lack of effect observed in this study is unlikely due to antagonist specificity and efficiency issues (Ogden and Traynelis, 2011). However, the involvement of NMDA receptors in mediating synaptic transmission within the ENS cannot yet be ruled out.

Despite considerable evidence for the expression of AMPA receptor subunits in rodents (Liu et al., 1997; Seifi and Swinny, 2016), we did not find robust effect of exogenous AMPA on myenteric neurons, or any effect of AMPA blockade on L-Glu evoked responses. However, the role of AMPA receptors in the ENS warrants future investigation.

The Group I mGluR antagonist (PHCCC), which targets both mGlu1 and mGlu5 receptor subtypes, did not alter [Ca^2+^]_i_ transients or the number of neurons responsive to exogenous L-Glu. It could be that the effect of exogenous glutamate is dominated by receptors other than group I mGluRs, and hence the antagonist was ineffective. However, 20 pulse train-evoked [Ca^2+^]_i_ transients were significantly reduced by PHCCC. This indicates that endogenous release of glutamate activates Group I mGluRs to regulate slow synaptic potentials. Moreover, it is likely that glutamate mediates slow transmission to calb+ myenteric neurons, as calb+ neurons receive substantial numbers of vGluT2+ varicosities and constitute the majority of L-Glu and 20 pulse responsive neurons. These observations are consistent with several electrophysiological studies that implicate Group I mGluRs in slow excitatory synaptic events in enteric neural circuitry (Liu and Kirchgessner, 2000; Ren et al., 2000; Foong and Bornstein, 2009). This suggests a role for both glutamate in mediating transmission within enteric circuits and for Group I mGluRs in the regulation of gastrointestinal function.

## Conclusion

We used anatomical and functional assays to demonstrate that glutamate excites calb+ neurons via the activation of Group I mGluRs. Further investigation of the group I mGluR subtypes involved could be fruitful in identifying the mechanism behind glutamatergic action on calb+ neurons, and the reflex circuitry underlying colonic motility.

## Data Availability

All datasets generated for this study are included in the manuscript and/or the supplementary files.

## Ethics Statement

This study was carried out in accordance with the recommendations of the ‘University of Melbourne Animal Experimentation Ethics Committee’. The protocol was approved by the ‘University of Melbourne Animal Experimentation Ethics Committee’.

## Author Contributions

MS, JB, and JF conceived and designed the experiments. MS and JF performed the experiments. MS analyzed the data. JB and JF contributed reagents, materials, and analysis tools. MS, EH-Y, and JF wrote the manuscript. All authors contributed to editing and revising the manuscript. All authors read and approved the final manuscript.

## Conflict of Interest Statement

The authors declare that the research was conducted in the absence of any commercial or financial relationships that could be construed as a potential conflict of interest.
